# The Hombres Saludables Physical Activity Web-Based and Mobile Phone Intervention: Pilot Randomized Controlled Trial With Latino Men

**DOI:** 10.2196/39310

**Published:** 2023-12-07

**Authors:** Akilah J Dulin, Shira Dunsiger, Tanya Benitez, Britta Larsen, Bess H Marcus, Gregory Champion, Kim M Gans

**Affiliations:** 1 Center for Health Promotion and Health Equity Brown University Providence, RI United States; 2 Herbert Wertheim School of Public Health and Human Longevity Science University of California San Diego, CA United States; 3 Department of Human Development and Family Sciences University of Connecticut Storrs, CT United States

**Keywords:** physical activity, Latino, Hispanic, men, eHealth, expert system, internet, SMS text messaging, mobile phone, social media, mobile health, mHealth, mobile phone

## Abstract

**Background:**

Owing to structural-level, interpersonal-level, and individual-level barriers, Latino men have disproportionately high rates of physical inactivity and experience related chronic diseases. Despite these disparities, few physical activity (PA) interventions are culturally targeted for Latino men.

**Objective:**

This study reported the feasibility and acceptability of Hombres Saludables PA intervention for Latino men. We also reported the preliminary efficacy of the intervention on PA change and provided the results of the exploratory moderator and mediator analysis.

**Methods:**

We completed a 6-month, single-blind, pilot randomized controlled trial of Hombres Saludables with Latino men aged between 18 and 65 years. Men were randomized to either (1) a theory-driven, individually tailored, internet-based and SMS text message–based, Spanish-language PA intervention arm or (2) a nutrition and wellness attention contact control arm that was also delivered via the web and SMS text message. We assessed the primary study outcomes of feasibility using participant retention and acceptability using postintervention survey and open-ended interview questions. We measured the preliminary efficacy via change in minutes of moderate to vigorous PA per week using ActiGraph wGT3X-BT accelerometry (primary measure) and self-reported minutes per week using 7-day Physical Activity Recall. Participants completed the assessments at study enrollment and after 6 months.

**Results:**

The 38 participants were predominantly Dominican (n=8, 21%) or Guatemalan (n=5, 13%), and the mean age was 38.6 (SD 12.43) years. Retention rates were 91% (21/23) for the PA intervention arm and 100% (15/15) for the control arm. Overall, 95% (19/20) of the intervention arm participants reported that the Hombres study was somewhat to very helpful in getting them to be more physically active. Accelerometry results indicated that participants in the intervention group increased their PA from a median of 13 minutes per week at study enrollment to 34 minutes per week at 6 months, whereas the control group participants showed no increases. On the basis of self-reports, the intervention group was more likely to meet the US PA guidelines of 150 minutes per week of moderate to vigorous PA at 6-month follow-up, with 42% (8/19) of the intervention participants meeting the PA guidelines versus 27% (4/15) of the control participants (odds ratio 3.22, 95% CI 0.95-13.69). Exploratory analyses suggested conditional effects on PA outcomes based on baseline stage of motivational readiness, employment, and neighborhood safety.

**Conclusions:**

The PA intervention demonstrated feasibility and acceptability. Results of this pilot study indicate that the Hombres Saludables intervention is promising for increasing PA in Latino men and suggest that a fully powered trial is warranted. Our technology-based PA intervention provides a potentially scalable approach that can improve health in a population that is disproportionately affected by low PA and related chronic disease.

**Trial Registration:**

ClinicalTrials.gov NCT03196570; https://classic.clinicaltrials.gov/ct2/show/NCT03196570

**International Registered Report Identifier (IRRID):**

RR2-10.2196/23690

## Introduction

In the United States, physical inactivity poses a serious problem to population health owing to its association with chronic diseases (eg, cardiovascular disease and type 2 diabetes), premature disability, and death [1-5]. However, regular performance of moderate to vigorous physical activity (MVPA) can prevent or delay the onset of many of these diseases and other adverse outcomes, even in the absence of clinically significant weight loss [2,3,6]. MVPA is associated with more positive mental health outcomes [7,8] and can be an effective disease prevention strategy. Therefore, owing to its implications for chronic disease prevention and disease risk reduction, increasing MVPA is a high priority of global and national health organizations, such as the World Health Organization and US Healthy People [9,10].

Despite the known health risks associated with physical inactivity, less than one-fourth of American adults meet the national MVPA guidelines [2,11]. A population subgroup with low MVPA levels and increased risk of chronic diseases is Latino men. In the United States, Latino people are the largest ethnic minority group [12], and they are burdened disproportionately by chronic diseases related to physical inactivity [13,14]. In 2018, approximately half of Latino men did not meet the national guidelines for either aerobic or muscle-strengthening activities [11]. Across 5 Latino men subgroups, Dominican and Cuban men had the lowest MVPA aerobic levels (28% and 35% met the recommendation, respectively), whereas Mexican American, Central American, and South American men were the most active (approximately 44% of each group met the recommendations) [15]. These findings indicate that specific subgroups of Latino men are at greater risk of being physically inactive and may have increased risks for chronic diseases associated with physical inactivity.

Latino men’s low levels of MVPA can be attributed in part to adverse social determinants of health (eg, structural inequities and occupation-related barriers) and to behavioral factors [16-18]. Often, these factors co-occur and interact to affect physical activity (PA) behaviors adversely. For instance, some Latino subgroups disproportionately live in neighborhoods with structural-level inequities such as low walkability, the lack of public recreation areas, and high crime rates, which may reduce opportunities for engaging in MVPA [15,19-21]. In fact, some Latino men report the lack of access to safe spaces as an important barrier to engagement in regular MVPA [17,18]. In addition, Latino men are overrepresented in occupations that involve physically taxing labor, and they commonly cite long work hours and fatigue as reasons for not performing MVPA [17,22,23]; this is concerning because occupation-based PA may not produce the same health benefits as MVPA [24]. Moreover, few programs delivered in Spanish, time constraints, competing time demands (eg, family commitments and work responsibilities), and transportation issues are barriers to Latino men’s participation in PA programs [18,22,23]. Thus, there is a need for PA interventions that circumvent the adverse social determinants of health, address behavioral barriers to PA, and meet the needs of diverse Latino men.

Results of previous interventions indicate that culturally and linguistically appropriate, individually tailored interventions can improve MVPA levels among Latina women [25,26]. However, Latino men are underrepresented in, and rarely targeted specifically in, interventions designed to increase MVPA levels. A previous systematic review of PA interventions with Latino adults found that 48% of the interventions included men and none of them specifically targeted men [27]. Of the 21 interventions reviewed, none included >36% Latino men in a single intervention [27]. In addition, many MVPA interventions with Latino adults include activities such as walking groups and dance classes, which are more likely to appeal to women [23,28]. Previous research indicates that phone-based interventions that incorporate SMS text messaging or a smartphone app component may be appealing to Latino men who are trying to adopt a healthy lifestyles, given the real constraints of adverse social determinants of health [17]. Thus, the use of technology in gender-appropriate and culturally appropriate interventions may help Latino men to engage in PA programs.

Considering the gaps in MVPA research described previously, we developed a primarily internet-based *Hombres Saludables* PA pilot randomized controlled trial (RCT) for diverse, Spanish-speaking Latino men (eg, Caribbean and South American). This intervention is based on our previous study with Latina women who participated in the successful RCT, *Pasos Hacia La Salud*, which led to increased PA levels sustained over a 12-month period [26,29].

The objectives of this study were to report feasibility (eg, retention) and acceptability (eg, participant satisfaction). As a secondary study outcome, we reported the preliminary efficacy of the intervention on PA and the potential moderators (eg, neighborhood environment) and mediators (eg, self-efficacy) of intervention efficacy. We will use the findings from this pilot intervention to inform the refinement and testing of the Hombres Saludables intervention in a fully powered RCT.

## Methods

As the Hombres Saludables formative study results, cultural and gender adaptations, participant recruitment, intervention protocol, evaluation methods and measures, and power calculations are published in extensive detail elsewhere [30], we have briefly described the intervention methods and measures in the subsequent sections.

### Study Design and Overview

The Hombres Saludables PA intervention used a single-blind RCT design. Spanish-speaking Latino men were randomized to either the intervention (ie, PA) or attention-matched control (ie, wellness and nutrition) condition using a permuted block randomization procedure and stratified randomization based on the Transtheoretical Model stages of change [31]. We used this randomization approach to ensure equal distribution of motivational readiness to adopt MVPA. The 6-month, individually tailored, internet-based and SMS text message–based intervention included evaluations at study enrollment and 6-month follow-up and extensive process evaluation data collection. The main PA outcome was minutes of MVPA per week (as measured using the accelerometer), and the secondary outcome was self-reported minutes of MVPA per week (as measured using 7-day Physical Activity Recall [PAR]) [32]. The RCT was registered with the clinical trials registry of the United States (ClinicalTrials.gov; NCT03196570). All intervention-specific recruitment and data collection activities occurred between 2018 and 2020.

### Ethical Considerations

All the study protocols received approval from the institutional review boards of the Brown University and University of Connecticut via an institutional review board authorization agreement (1612001664). All study participants provided written informed consent after review of study consent forms with program staff. Participants received up to US $215 as participant incentives for study participation and gym membership worth US $60 (or financial equivalent). Participants provided identifiable data which were stored on secure Brown University network servers. Only authorized personnel had access to participants identifiable data.

### Participant Recruitment and Eligibility

Spanish-speaking staff used a variety of active and passive recruitment methods in the New England area (Rhode Island, Connecticut, and Massachusetts) and nationally. In the New England area, staff actively recruited at businesses, local libraries, churches, and colleges. In addition, staff conducted outreach to men’s groups, worksites, and community organizations. They also posted flyers and completed interviews and paid radio advertisements on Spanish-language radio. Although these efforts resulted in high reach among Latino men locally, study enrollment lagged. As such, we expanded study recruitment beyond New England by posting paid advertisements on social media sites (eg, Facebook), with specific focus on recruiting Latino men from the East Coast of the United States.

Staff determined the initial study eligibility via a phone screening with men interested in learning more about the study. To be eligible, potential participants had to self-report being a Latino man aged between 18 and 65 years. As the intervention was primarily internet-based and SMS text message–based, potential participants also had to own a mobile phone with SMS texting capabilities and have regular internet access via a smartphone, tablet, or computer. Potential participants had to agree to be randomized to either the intervention or attention-matched control arm. As part of the preliminary eligibility screening, participants answered questions about medical conditions to identify conditions that might increase the health risks of engaging in unsupervised MVPA. Additional exclusion criteria included BMI≥45 kg/m^2^, hospitalization owing to a psychiatric disorder in the past 3 years, planned surgery or hospitalization in the next 6 months, and taking medication that may negatively affect the person’s ability to engage in MVPA. Staff referred any health-related eligibility concerns to the Hombres Saludables study physician for eligibility determination.

### Visits to Confirm Eligibility, Enrollment, and Study Randomization

Latino men who resided in Rhode Island and Massachusetts attended in-person visits at Brown University, whereas men outside this geographic area completed remote visits using a combination of phone, internet, or mailed materials.

#### Visit 1: Confirmation of Study Eligibility and Completion of Surveys Upon Study Enrollment

Potential participants completed additional eligibility screening including a questionnaire to demonstrate Spanish-language literacy (ie, score ≥16 on the Spanish-language Short-Test of Functional Health Literacy in Adults) [33] and low PA (ie, self-reports of ≤60 min of MVPA per week measured in 10-min bouts and excluding occupation-related PA) using 7-day PAR [32]. If eligibility was met, the study staff explained the consent forms and addressed all participants’ questions; interested participants then provided written informed consent. After completing study consent procedures, staff measured participants’ height, weight, and waist circumference (in person only). Remote participants provided self-reports of height and weight only. Men with BMI≥45 kg/m^2^ were ineligible at this stage. Participants completed questionnaires about demographics and PA-related psychosocial variables and an internet and SMS text messaging accessibility check. Participants received an ActiGraph wGT3X-BT accelerometer, instructions for its placement around the waist and wear time for 7 consecutive days, and a form to log accelerometer wear dates and times.

#### Visit 2: Randomization

This visit was scheduled approximately 8 days after visit 1. All (ie, in person and remote) participants returned the worn accelerometer and PA log form to the study staff. For participants who did not meet the wear-time criterion (<3000 min over 4 d or <5 d of 600 min/d), the study staff asked them to rewear the accelerometer and then rescheduled visit 2. To familiarize them with moderate PA and improve the accuracy of their self-reports of PA during the 6 months of the study, in-person participants completed a 10-minute walk test (treadmill set at 3-4 miles/h) [25]. These participants reported their rate of perceived exertion, and the staff collected heart rate measures. For remote participants, the staff described MVPA and provided examples of activities. All participants completed another 7-day PAR and had to meet the eligibility criterion for this measure, as described in the previous section [32].

After completing the visit-2 activities, participants were randomized to either the tailored PA intervention arm or the nutrition and wellness control arm. To randomize participants, the research assistant (RA) reviewed the participant’s data to identify the participant’s stage of change and then selected the randomization envelope corresponding to that specific stage of change. Hombres participants who reported that a family or household member was enrolled in another PA study conducted by our research team were yoked during randomization to the same study arm of the family or household member to prevent cross-treatment contamination. After randomization, the RA helped the participants set up their website accounts and guided them through the website. In addition, the RA helped the participants set up personalized exercise goals, described the PA plans for their first week, and supported participants as they entered their information into the study website.

### Intervention Content

The theory-based Hombres Saludables intervention arm consisted of several components, including an intervention website, SMS text messages, private Facebook group, gym membership, and check-in phone calls. The behavior change strategies (eg, goal setting, problem-solving, and stages of change) were informed by the Social Cognitive Theory and the Transtheoretical Model [31,34]. The intervention components that mapped onto each specific theoretical construct have been described in a protocol paper [30]. The intervention website included Spanish-language resources to promote PA (eg, exercise videos, PA apps, city guides, and PA tip sheets). In addition, participants used the website to log their MVPA each day, to set and log PA goals each week, and to complete monthly surveys that measured Social Cognitive Theory and Transtheoretical Model constructs. On the basis of the questionnaire results, participants received tailored PA reports and manuals that included feedback based on their motivational readiness for PA and self-efficacy and cognitive and behavioral strategies to increase PA. In addition, the reports provided participants with PA norm information—how physically active they were compared with other adults and their previous PA level. We adapted the website and tailored reports from the Pasos Hacia La Salud intervention [26,29].

Participants in the intervention arm received 4 to 6 autogenerated SMS text messages each week. These SMS text messages included reminders to engage with the website intervention materials and activities, content related to social support, and suggestions to overcome PA barriers. In addition, participants could opt in to a private Facebook group that contained PA tips, events, and information. Moreover, participants could use the Facebook page to provide social support to one another. To encourage participation on the Facebook page, engaged participants were entered into a random prize drawing (US $25) conducted monthly. Participants from the New England area received a 6-month voucher for membership at a local gym franchise (or financial equivalent of US $60 for distant participants). Participants received a check-in phone call at 1 week and 1 month after study enrollment. During these calls, study staff ensured that participants received study materials and could use the internet resources, discussed goal setting, and answered participants’ questions.

### Control Group Content

The nutrition and wellness control group was an attention-matched control with participants also receiving access to a Spanish-language website, SMS text messages, a private Facebook page, and check-in phone calls. The website contained nutrition-related and wellness-related resources (eg, tip sheets and healthy eating recipes). Participants completed monthly surveys on the website (eg, diet and sleep) and received up to 4 SMS text messages that contained website reminders and tips. Participants could opt in to a private Facebook page that included additional topics. Finally, they received check-in phone calls 1 week and 1 month after study enrollment.

### Measures

The measures used are summarized briefly. For more details about specific question wording used in the measures and items, please refer to the paper by Gans et al [30].

Primary study outcomes were feasibility and acceptability. Although we measured feasibility using several approaches (eg, proportion of recruited participants eligible to participate in the study and recruitment method yields), we defined the primary measure as participant retention of ≥80% over the course of the intervention. At the end of the intervention, participants completed follow-up surveys that included questions about acceptability. Specifically, participants reported their level of satisfaction with each intervention component; frequency that each intervention component was read, used, or accessed; helpfulness of each intervention component; and satisfaction with the intervention overall. We also conducted poststudy interviews with a subset of participants.

Secondary outcomes of objective and subjective MVPA were assessed at study enrollment and 6-month follow-up. To objectively measure PA, participants wore an accelerometer (ActiGraph wGT3X-BT). Although the staff instructed participants to wear the accelerometer for 7 days, minimum acceptable wear time was either 5 days with ≥600 minutes per day or 4 days with ≥3000 total minutes. To determine MVPA, we used an established minimum cutoff of 1952 counts per minute and a minimum bout of 10 minutes of PA [35-37]. As mentioned previously, participants self-reported PA using 7-day PAR. Trained staff members certified in the 7-day PAR protocol queried participants about their moderate, hard, and very hard physical activities during specified periods (eg, morning and afternoon) each day for the past 7 days [38,39]. The 7-day PAR has demonstrated reliability and concurrent validity with objective measures of PA and is sensitive to changes in PA over time [32,40-42].

Characteristics of the neighborhood social and built environment were explored as potential moderators of intervention efficacy. Participants self-reported their perceptions about neighborhood safety and neighborhood social cohesion [43,44]. Participants also completed several measures related to policing including perceived neighborhood police attitudes or policing, fear of police, and fear of police profiling while being physically active [45-47]. Objective built environment features related to PA were assessed. Trained staff members completed internet-based built environment audits of participants’ neighborhood environments using a Google Street View protocol and the Active Neighborhood Checklist. Built environment audits via Google Street View, including the Active Neighborhood Checklist, have demonstrated reliability with in-person neighborhood audits [48-52].

We explored psychosocial variables as potential mediators of intervention efficacy. At study enrollment and 6-month follow-up, participants completed questionnaires about readiness to change PA (eg, precontemplation and contemplation) [53,54], processes of PA change to assess cognitive and behavioral strategies [55], and self-efficacy for PA [53]. In addition, participants completed these psychosocial measures monthly via the Hombres Saludables website; these monthly data points were used to develop tailored feedback to support PA change among intervention participants. Additional psychosocial measures included social support for PA from friends and family over the past 3 months and the Perceived Stress Scale (eg, level of stress in the past month) [56,57].

### Demographics

At study enrollment, participants completed demographic questions about age, education, employment status, income, and marital status. Participants also completed questions related to Hispanic subgroup (eg, Puerto Rican or Cuban), language acculturation (using the Brief Acculturation Scale) [58], and years lived in the United States.

### Analysis

Baseline sociodemographics and PA level at study enrollment were summarized for each condition, and descriptive statistics are reported. Correlation analysis was used to explore potential confounding effects of the intervention. Although interest was not in strict statistical hypothesis testing, a modest benchmark of *P*<.30 was used to identify potential confounders that should be adjusted for in subsequent models of PA outcomes.

As the primary study aim was to determine the feasibility and acceptability of the intervention, retention rates at the end of treatment were compared between conditions using chi-square tests, and participants’ satisfaction was summarized and compared between groups. Hypothesis testing was used in this case to examine between-group differences in these metrics.

The secondary outcome of interest was minutes per week of MVPA. Graphical methods suggested that PA data were skewed, and transformations toward normality were not successful (data were still skewed following log transformation). As such, quantile regression was used to estimate the effects of intervention versus control conditions on median minutes per week of MVPA. Models controlled for value of the outcome at study enrollment; employment (which differed significantly between groups and was significantly correlated with MVPA outcomes); and in the case of objectively measured MVPA, accelerometer wear time. As a subsequent step, we explored the potential conditional effects of key demographic and neighborhood-level variables to better understand the potential moderators of the treatment effect using a similar analytic approach. Models included the main effects of the condition and interactions with the potential moderator.

Next, using a generalized linear model with logit link function, we examined the effects of treatment condition (intervention vs control condition) on the odds of meeting national guidelines for PA (defined as at least 150 min/wk of MVPA). Interest was in estimating the odds ratios and the corresponding CIs. Models were adjusted for employment (confounder identified a priori). Using a similar analytic approach (quantile regression and generalized linear model), we explored the effects of dose on treatment outcomes (min/wk of MVPA and meeting national guidelines) within the intervention condition. In all cases, interest was not in determining statistical significance but rather in estimating effect sizes for a future fully powered study.

Although limited by sample size, to generate hypotheses related to mechanisms of the treatment, a product-of-coefficients approach with bootstrapped SEs (10,000 bootstrapped samples) was used. Given the pilot nature of this study, the focus was on univariate mediation models and estimating path coefficients (effects of treatment condition on changes in the mediators [a path], effects of changes in mediators on PA outcomes [b path], and the indirect effect of the intervention [ab path]).

In all cases, models used a likelihood or quasi-likelihood–based approach to estimation and thus used all available data (on the intent to treat sample) without directly imputing missing outcomes. All analysis was performed using SAS 9.4 (SAS Institute Inc), with significance level set at .05 a priori.

## Results

### Recruitment Yield

**Figure 1:**
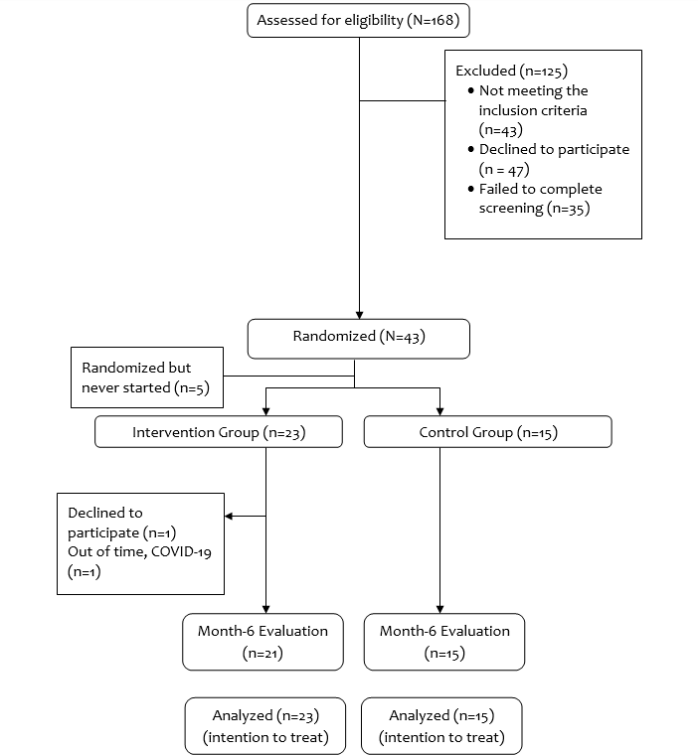
Hombres Saludables CONSORT (Consolidated Standards of Reporting Trials) flow diagram.

A total of 168 men expressed interest in the study during initial recruitment (refer to the CONSORT [Consolidated Standards of Reporting Trials] diagram in Figure 1). Of these 168 men, 43 (25.6%) were ineligible, 47 (28%) declined to participate, and 35 (20.8%) did not complete the screening. The remaining 25.6% (43/168) of the men were randomized. After randomization, 12% (5/43) of the men never started the intervention. Thus, we ended up with a total of 38 men in the pilot trial, including 23 (61%) in the intervention group and 15 (39%) in the control group.

### Characteristics of the Study Participants

Th 38 participants were predominantly Dominican (n=8, 21%) or Guatemalan (n=5, 13%), and the mean age was 38.6 (SD 12.43) years. Approximately two-thirds (24/38, 63%) reported at least some college education, and 58% (22/38) were employed full time. A full description of the study sample is presented in Table 1.

**Table 1:** Sample descriptive statistics categorized according to study arm for Latino men participating in the pilot trial of Hombres Saludables (n=38).

Characteristics				Physical activity intervention group (n=23)		Nutrition and wellness control group (n=15)
**Sociodemographic data**						
	Age (18-61 y), mean (SD)		39.39 (11.89)		37.40 (13.57)	
	BMI (20.4-44.4 kg/m^2^), mean (SD)		30.47 (6.41)		30.46 (4.82)	
	**Nationality, n (%)**					
		Puerto Rican	1 (4)		1 (7)	
		Dominican	7 (30)		1 (7)	
		Mexican or Mexican American	2 (9)		1 (7)	
		Cuban	0 (0)		0 (0)	
		Guatemalan	2 (9)		3 (20)	
		Colombian	2 (9)		1 (7)	
		Salvadoran	1 (4)		0 (0)	
		>1 subgroup	4 (17)		3 (20)	
		Other	4 (17)		5 (33)	
	**Race, n (%)**					
		American Indian or Alaska Native	0 (0)		2 (13)	
		Black	1 (4)		0 (0)	
		White	8 (35)		5 (33)	
		>1 race	5 (22)		5 (33)	
		Other	8 (35)		3 (20)	
	**Education, n (%)**					
		Less than high school	3 (13)		5 (33)	
		High school or GED^a^	1 (4)		1 (7)	
		Vocational or technical	3 (13)		1 (7)	
		At least some college	16 (70)		8 (53)	
	**Income (US $), n (%)**					
		<10,000	2 (9)		3 (20)	
		10,000-20,000	3 (13)		3 (20)	
		20,000-30,000	7 (30)		2 (13)	
		30,000-40,000	7 (30)		3 (20)	
		40,000-50,000	0 (0)		1 (7)	
		≥50,000	4 (17)		3 (20)	
	**Employment, n (%)**					
		Unemployed	1 (4)		5 (33)	
		Full time	15 (65)		7 (47)	
		Part time	7 (30)		3 (20)	
	**Marital status, n (%)**					
		Never married	7 (30)		6 (40)	
		Divorced	2 (9)		1 (7)	
		Widowed	0 (0)		1 (7)	
		Married	14 (61)		6 (40)	
		Living with partner	0 (0)		1 (7)	
	**Neighborhood safety: do you feel safe in your neighborhood? n (%)**					
		All the time	6 (26)		8 (53)	
		Most of the time	14 (61)		5 (33)	
		Some of the time	2 (9)		2 (13)	
		None of the time	1 (4)		0 (0)	
	S-TOFHLA^b^, mean (SD; range)		32.78 (4.80; 17-37)		29.47 (7.35; 17-37)	
**Psychosocial data**						
	**Stage of change, n (%)**					
		Precontemplation	1 (4)		1 (7)	
		Contemplation	12 (52)		8 (53)	
		Preparation	9 (39)		6 (40)	
		Action	1 (4)		0 (0)	
	Behavioral processes, mean (SD; range)		2.86 (0.51; 1.4-4.2)		2.97 (0.73; 1.4-4.2)	
	Cognitive processes, mean (SD; range)		3.20 (0.51; 1.8-4.6)		3.11 (0.72; 1.8-4.6)	
	Self-efficacy, mean (SD; range)		2.77 (0.76; 1.4-4.6)		2.65 (0.76; 1.4-4.6)	
	Perceived stress, mean (SD; range)		14.65 (5.21; 2-23)		12.07 (4.45; 2-23)	
	**Social support, mean (SD; range)**					
		Family participation	16.95 (6.52; 10-37)		16.93 (7.24; 10-37)	
		Friend participation	15.18 (4.25; 10-29)		17.07 (7.35; 10-29)	
		Family reward	4.13 (2.07; 3-10)		3.87 (0.92; 3-10)	
**Physical activity level, mean (SD; median)**						
	**Objectively measured min/wk of MVPA^c^**					
		Values, mean (SD)	57.83 (100.80)		35.20 (49.32)	
		Values, median (range)	13 (0-299)		22 (0-299)	
	**Self-reported min/wk of MVPA**					
		Values, mean (SD)	93.83 (250.10)		86.47 (158.64)	
		Values, median (range)	0 (0-1140)		0 (0-1140)	

^a^GED: general education diploma.

^b^S-TOFHLA: Spanish-language Short-Test of Functional Health Literacy in Adults—a questionnaire to assess functional literacy.

^c^MVPA: moderate to vigorous physical activity.

### Feasibility and Acceptability

Retention rates were 91% (21/23) for the intervention arm and 100% (15/15) for the control arm, suggesting that the intervention is feasible. Regarding acceptability, 85% (18/21) of the intervention participants reported that the Hombres staff were helpful or very helpful and 90% (18/20) of the participants reported that the research study was at least somewhat helpful to very helpful. Overall, 75% (16/21) of the intervention group participants reported that they gained some to a lot of knowledge from the study website and 100% (20/20) reported that they were somewhat to very motivated from using the website. Overall, 95% (19/20) of the participants reported that the Hombres study was somewhat to very helpful in getting them to be more physically active. All the intervention participants (21/21, 100%) reported that they were somewhat to very satisfied with the Hombres study, and all (20/20, 100%) reported that they would recommend it to a friend.

Participants completed follow-up surveys with open-ended qualitative questions; moreover, we conducted postintervention qualitative interviews with 76% (16/21) of the PA intervention participants and 53% (8/15) of the control group participants. The interview results indicated that participants were happy with the study enrollment process, but thought that the enrollment process was long, and a few of the men had language difficulties and would have preferred the intervention in English. For example, an interview participant said the following:

Those surveys should be checked. They’re too long...

Another participant said the following:

...The language – because...those who are born here have difficulty speaking Spanish.

In terms of the intervention, participants viewed the website and the SMS text messages favorably, but the Facebook intervention was used less often by participants. Participants in the PA group indicated that the intervention provided them with *the emotional support, and the support to keep us physically active* and *the motivation to exercise and the resources to make it happen*. Participants in the control group liked the nutrition-related advice and tips. In terms of ideas for future interventions, the men indicated that they would prefer an activity monitor worn on the wrist rather than the waist. They also recommended joining a future study together with a partner such as a spouse. For example, interview respondents said the following:

Because, if you don’t do it, the spouse or partner motivates you...I think that doing it alone is the difficult part of the program.

Participants also wanted more interaction with other participants and group activities in future interventions. A PA group participant said the following:

If for example, there were an opportunity to gather all of us...so that you can go out to walk or do activities together...that could’ve helped a lot...

Participants also suggested having more personalized advice. Participants in the PA group expressed interest in future interventions that included nutrition. A participant said the following:

I believe that exercise is an essential part of being healthy, but what is really important is to maintain a healthy diet and help yourself with exercise.

### Process Evaluation

Objective website data indicated that PA intervention participants logged on to the study website an average of 46.44 (SD 47.72) times over 6 months compared with 27 (SD 20.94) times for wellness participants. Overall, participants in both conditions spent an average of 98.29 (SD 93.01) minutes over 6 months on the website, with a range of 10 to 541 minutes. For the entire duration of the intervention, PA intervention participants spent an average of 567.52 (SD 1465.31) minutes on the tips page and 3582.10 (SD 2866.99) minutes in reporting their activity. These participants also spent 49.07 (SD 117.74) minutes on the *web activity level* feature, 8.29 (SD 26.49) minutes on the *did you know* feature, and 25.19 (SD 65.41) minutes on the *expert* feature.

Overall, 95% (20/21) of the PA intervention participants completed the satisfaction surveys. Almost all (18/20, 90%) reported that recording their PA minutes on the website was helpful or very helpful. Similarly, 90% (18/20) reported that the website was at least somewhat helpful in helping them to set their goals. Overall, 85% (17/20) reported that the website message board was at least somewhat helpful, and 85% (17/20) reported that the *ask the expert* feature was at least somewhat helpful. Finally, 95% (19/20) of the participants reported that the *ways to be active* feature was at least somewhat helpful.

All participants (20/20, 100%) reported that the SMS text messages were at least somewhat helpful; 50% (10/20) reported them to be very helpful. Overall, 80% (16/20) of the respondents reported that the tips about exercise were the most helpful type of SMS text messages received (with 4/20, 20% reporting that the reminders to use the website were the most helpful). Of the 20 participants, 2 (10%) reported that they read all the tips sent via SMS text message, 3 (15%) read most of them, 4 (20%) read half of them, 8 (40%) read a few of them, and 2 (10%) read none. Overall, 95% (19/20) of the participants reported that the SMS text message reminders to engage with the study website were at least somewhat helpful.

Overall, 50% (10/20) reported reading at least half of the tip sheets. When asked about their use of the Facebook page, 25% (5/20) used it at least 1 to 2 days per week. In addition, 50% (10/20) of the participants reported using their Hombres gym membership at least 1 to 2 days per week.

### PA Outcomes

The secondary outcome results for changes in PA are presented in Table 2. As this was a pilot study not powered on PA outcomes, the following findings should be interpreted with caution, especially given the wide CIs presented in the statistical analysis.

**Table 2:** Objectively measured and self-reported physical activity outcomes categorized according to group, from study enrollment to 6-month follow-up among Hombres Saludables intervention participants.

Variable	Physical activity intervention group	Nutrition and wellness control group
Change in median objectively measured MVPA^a^/wk (baseline to 6 mo), min/wk	13-34	22-22
Change in median self-reported MVPA/wk (baseline to 6 mo), min/wk	0-150	0-30
Proportion meeting ACSM^b^ guidelines of ≥150 min/wk of MVPA at 6 mo, n (%)	8 (42.1)^c^	4 (26.7)^d^

^a^MVPA: moderate to vigorous physical activity.

^b^ACSM: American College of Sports Medicine.

^c^Sample size, n=19.

^d^Sample size, n=15.

For the intervention arm, objectively measured minutes per week of MVPA increased from a median of 13 (IQR 0-57) minutes per week to 34 (IQR 0-107.5) minutes per week at 6 months (Table 2). However, for the control group participants, median minutes per week of MVPA was 22 (IQR 0-47 at study enrollment and IQR 0-42 at 6 months) at both study enrollment and 6 months. Point estimates from the adjusted model indicated that the intervention arm participants had high median minutes per week of MVPA at 6 months versus control arm participants (b=34, SE 17.61, 95% CI −0.52 to 68.52).

Intervention participants increased their self-reported MVPA from a median of 0 (IQR 0-44.75) minutes per week at study enrollment to 105 (IQR 0-325) minutes per week at 6 months, whereas control participants increased their self-reported MVPA from 0 (IQR 0-60) minutes per week at study enrollment to 30 (IQR 0-152) minutes per week at 6 months. Point estimates from the quantile regression models suggested differences in 6-month outcomes between conditions controlling for study enrollment (b=105, SE 76.14, 95% CI –44.23 to 254.23).

Similarly, based on self-reported data, there were trends suggesting that a high proportion of intervention participants met the national guidelines for PA (defined as reporting at least 150 minutes per week of MVPA) [2,3] at 6 months (odds ratio 3.22, 95% CI 0.95-13.69). Overall, 42% (8/19) of PA intervention participants met the criteria at 6 months compared with 27% (4/15) of the control participants.

### Dose Effects

Among intervention participants, a high dose of intervention received (defined as total engagement with the study website) was associated with greater self-reported MVPA outcome at 6 months (*f*^2^=0.14) and increased odds of meeting national PA guidelines (*f*^2^=0.12). Furthermore, among those randomized to the intervention arm, higher MVPA rates at end of treatment were seen for those with greater engagement with the study website (more log-ins, higher frequency of goal setting, and higher frequency of PA tracking) when controlling for baseline PA.

### Moderators of Intervention Effect

Exploratory moderator analyses indicate that stage of motivational readiness, employment, and neighborhood safety may serve as moderators of the intervention effect on PA outcomes. Intervention participants in the contemplation stage reported more minutes per week of MVPA at 6 months versus control participants (b=160, SE 73.20). Among participants who were not employed full time, PA participants outperformed control participants at 6 months (b=195, SE 94.26). Intervention participants who reported feeling safe in their neighborhood all or most of the time, reported greater PA at 6 months versus control participants (b=60, SE 33.43). Additional neighborhood-level variables (social cohesion, police attitudes, fear of police, police attitudes as a barrier, and built environment audit summary score) did not appear to moderate the intervention effects in this pilot study.

### Mediator Models

Exploratory analyses provided some indication that self-efficacy may mediate the intervention effect. Intervention participants increased their self-efficacy more than control participants (a=0.54, SE 0.23), and these increases in self-efficacy were associated with more minutes per week of MVPA at 6 months (b=178.65, SE 87.59).

## Discussion

### Principal Findings

The objectives of this study were to demonstrate the feasibility, acceptability, and preliminary efficacy of the pilot RCT, Hombres Saludables trial, which was a 6-month PA eHealth intervention for diverse Spanish-speaking Latino men. Overall, the intervention demonstrated feasibility, with a very high retention rate (>90%). The participants indicated that the intervention was acceptable in the follow-up survey and qualitative interviews. PA participants frequently used the website and its individual features and reported satisfaction with these intervention components. The intervention group increased their levels of MVPA (measured objectively and subjectively) relative to the control group. However, as this was a pilot study, we did not have the power to detect statistical significance. Some of the intervention moderators (eg, neighborhood safety) and a mediator (ie, self-efficacy) demonstrated some effects on the intervention. Although the preliminary efficacy findings were promising, these should be interpreted with caution given the very small sample size for this pilot study and the wide CIs. However, the point estimates were in the right direction, which suggests that a fully powered trial is warranted.

This study’s findings were consistent with previous pilot feasibility and acceptability PA or weight management interventions with Latino men [28,59-61]. Many of these studies demonstrated that recruitment was feasible, and once men were enrolled in the intervention, retention was very high. For example, in a similar tailored, print-based intervention of the same duration, approximately 93% of men were retained at 6-month follow-up [60]. In addition, findings from other pilot interventions suggested that Latino men were satisfied with PA interventions and that they were helpful in promoting or increasing engagement in PA [59,61]. These findings underscore the need to provide PA-specific resources and interventions to Latino men.

Preliminary efficacy results suggested that intervention participants increased their levels of PA more than control group participants. For instance, similar increases in meeting PA guidelines after 6 months were observed in a pilot, Spanish-language PA intervention with Mexican American men and in a Spanish-language PA intervention for Latina women [28,60]. These results suggest that a theory-based, Spanish-language, mobile health intervention delivered mainly via the internet may be a successful approach for increasing MVPA in Latino men. A fully powered randomized trial is needed to confirm the preliminary efficacy findings of this intervention.

Changes in PA appeared to relate directly to intervention engagement. Specifically, participants receiving higher dose of intervention self-reported more MVPA and were more likely to meet the United States PA guidelines. These results were consistent with those of previous studies. A recent systematic review and meta-analysis reported that engagement with PA digital health interventions had weak but positive associations with PA outcomes [62]. Specific PA intervention engagement components that were promising included measures of subjective experience, the number of PA intervention activities completed, and the total number of log-ins [62]. Thus, our finding of high engagement with the internet-based intervention is encouraging. Linke et al [63] reported that greater use of intervention components (ie, goal setting, personal PA reports, and PA tips) was positively associated with increase in MVPA at 12 months among primarily Mexican American women. The high levels of engagement in the study by Linke et al [63] and our Hombres Saludables trial bode well for future internet-based and SMS text-based interventions with Latino populations. A future fully powered trial of the Hombres Saludables study should include measures to determine which aspects of the intervention promote more engagement and are associated with PA outcomes.

In exploratory analyses, several moderators (motivational readiness or stage of change, employment, and neighborhood safety) and a mediator (self-efficacy) were associated with intervention outcomes. Intervention group participants in the contemplative stage of change (ie, those who did not engage in regular PA) reported higher minutes per week of MVPA at 6 months than control participants, which provides support for the intervention in increasing PA. The finding that participants in the PA intervention group who were not employed full time also reported significantly greater PA than those in the control group could be attributed in part to having additional time for MVPA and less work-related fatigue from working in physically demanding jobs [17,22,23]. PA participants who reported feeling safe all or most of the time in their neighborhoods reported great PA; this finding supports evidence from previous studies regarding the lack of safe neighborhood spaces for PA as a barrier [17,18]. Although informative, these exploratory findings should be interpreted with caution. Self-efficacy emerged as the only mediator of the intervention’s effect. Specifically, participants in the PA intervention group increased their self-efficacy more than the control group, and increases in self-efficacy were associated with high MVPA at 6 months. This finding is consistent with previous studies indicating that self-efficacy is one of the strongest predictors of PA in Latinos and reinforces the need to target this construct in PA interventions [64-66]. Future studies should examine these potential moderators and mediators in fully powered trials.

### Study Limitations

Although informative, this study is not without some limitations. One of the grant-approved aims of this pilot study was to assess preliminary efficacy. However, there have been growing calls to omit preliminary efficacy from pilot trials because they are very underpowered to detect effects, and the limited utility of these imprecise estimates could bias conclusions about the potential directions for future studies [67]. Thus, this study’s preliminary efficacy results should be interpreted with caution, especially given the wide CIs as noted in the *Results* section. Moreover, three-fourths (125/168, 74.4%) of the recruited participants declined to enroll in the study or stopped communicating with the staff. It is possible that characteristics of men who enrolled and participated differed from those of men who did not enroll, which in turn may have yielded more favorable study outcomes.

### Conclusions and Future Directions

The individually tailored, internet-based and SMS text messaging–based Hombres Saludables PA intervention demonstrated feasibility and acceptability with promising results for increasing MVPA among Latino men. Thus, a future, fully powered RCT is warranted. This technology-based PA intervention provides a potentially scalable approach that could improve PA and health in a population that is disproportionately affected by low PA and related chronic diseases. Future interventions should consider adding components to address neighborhood safety, including spouses or other family members, and involving more interaction with other participants. Future studies should also consider offering the intervention in both English and Spanish to reach Latino men with a wide range of acculturation levels, adding nutrition content, exploring mediation, and conducting implementing studies to determine the most effective intervention components.

## Appendix

Multimedia Appendix 1 CONSORT-eHEALTH checklist (V 1.6.1).

## Abbreviations

CONSORT: Consolidated Standards of Reporting Trials
MVPA: moderate to vigorous physical activity
PA: physical activity
PAR: Physical Activity Recall
RA: research assistant
RCT: randomized controlled trial

## Data Availability

The data sets generated during and analyzed during this study are not publicly available due to the pilot nature of this research study but may be available from the corresponding author via an institutional data use agreement.

## References

[1] Durstine JL, Gordon B, Wang Z, Luo X (2013). Chronic disease and the link to physical activity. J Sport Health Sci.

[2] (2008). 2008 Physical activity guidelines for Americans. U.S. Department of Health and Human Services.

[3] (2008). Physical activity guidelines advisory committee report, 2008. U.S. Department of Health and Human Services.

[4] Mokdad AH, Ballestros K, Echko M, Glenn S, Olsen HE, Mullany E, Lee A, Khan AR, Ahmadi A, Ferrari AJ, Kasaeian A, Werdecker A, Carter A, Zipkin B, Sartorius B, Serdar B, Sykes BL, Troeger C, Fitzmaurice C, Rehm CD, Santomauro D, Kim D, Colombara D, Schwebel DC, Tsoi D, Kolte D, Nsoesie E, Nichols E, Oren E, Charlson FJ, Patton GC, Roth GA, Hosgood HD, Whiteford HA, Kyu H, Erskine HE, Huang H, Martopullo I, Singh JA, Nachega JB, Sanabria JR, Abbas K, Ong K, Tabb K, Krohn KJ, Cornaby L, Degenhardt L, Moses M, Farvid M, Griswold M, Criqui M, Bell M, Nguyen M, Wallin M, Mirarefin M, Qorbani M, Younis M, Fullman N, Liu P, Briant P, Gona P, Havmoller R, Leung R, Kimokoti R, Bazargan-Hejazi S, Hay SI, Yadgir S, Biryukov S, Vollset SE, Alam T, Frank T, Farid T, Miller T, Vos T, Bärnighausen T, Gebrehiwot TT, Yano Y, Al-Aly Z, Mehari A, Handal A, Kandel A, Anderson B, Biroscak B, Mozaffarian D, Dorsey ER, Ding EL, Park E, Wagner G, Hu G, Chen H, Sunshine JE, Khubchandani J, Leasher J, Leung J, Salomon J, Unutzer J, Cahill L, Cooper L, Horino M, Brauer M, Breitborde N, Hotez P, Topor-Madry R, Soneji S, Stranges S, James S, Amrock S, Jayaraman S, Patel T, Akinyemiju T, Skirbekk V, Kinfu Y, Bhutta Z, Jonas JB, Murray CJ, US Burden of Disease Collaborators (2018). The state of US health, 1990-2016: burden of diseases, injuries, and risk factors among US states. JAMA.

[5] Carlson SA, Adams EK, Yang Z, Fulton JE (2018). Percentage of deaths associated with inadequate physical activity in the United States. Prev Chronic Dis.

[6] Martinez-Gomez D, Lavie CJ, Hamer M, Cabanas-Sanchez V, Garcia-Esquinas E, Pareja-Galeano H, Struijk E, Sadarangani KP, Ortega FB, Rodríguez-Artalejo F (2019). Physical activity without weight loss reduces the development of cardiovascular disease risk factors - a prospective cohort study of more than one hundred thousand adults. Prog Cardiovasc Dis.

[7] White RL, Babic MJ, Parker PD, Lubans DR, Astell-Burt T, Lonsdale C (2017). Domain-specific physical activity and mental health: a meta-analysis. Am J Prev Med.

[8] Rizvi S, Khan AM (2019). Physical activity and its association with depression in the diabetic hispanic population. Cureus.

[9] (2018). Global action plan on physical activity 2018–2030: more active people for a healthier world. World Health Organization.

[10] (2020). Physical activity. Office of Disease Prevention and Health Promotion.

[11] National health interview survey. Centers for Disease Control and Prevention.

[12] Quick facts. United States Census Bureau.

[13] Holmes JS, Madans JH (2013). Health, United States, 2012; with special feature on emergency care. Centers for Disease Control and Prevention.

[14] Profile: Hispanic/Latino Americans. Office of Minority Health Resource Center.

[15] Murillo R, Echeverria S, Vasquez E (2016). Differences in neighborhood social cohesion and aerobic physical activity by Latino subgroup. SSM Popul Health.

[16] Abraído-Lanza AF, Flórez KR, Shelton RC (2016). Acculturation and physical activity among Latinos. The Oxford Handbook of Acculturation and Health.

[17] Garcia DO, Valdez LA, Hooker SP (2017). Hispanic male's perspectives of health behaviors related to weight management. Am J Mens Health.

[18] Casper JM, Harrolle MG (2013). Perceptions of constraints to leisure time physical activity among Latinos in Wake County, North Carolina. Am J Health Promot.

[19] Murillo R, Reesor LM, Hernandez DC, Obasi EM (2019). Neighborhood walkability and aerobic physical activity among latinos. Am J Health Behav.

[20] Sallis JF, Floyd MF, Rodríguez DA, Saelens BE (2012). Role of built environments in physical activity, obesity, and cardiovascular disease. Circulation.

[21] Silfee VJ, Rosal MC, Sreedhara M, Lora V, Lemon SC (2016). Neighborhood environment correlates of physical activity and sedentary behavior among Latino adults in Massachusetts. BMC Public Health.

[22] Larsen BA, Noble ML, Murray KE, Marcus BH (2014). Physical activity in Latino men and women: facilitators, barriers, and interventions. Am J Lifestyle Med.

[23] Valdez LA, Morrill KE, Griffith DM, Lindberg NM, Hooker SP, Garcia DO (2019). Mexican origin Hispanic men's perspectives of physical activity-related health behaviors. Am J Mens Health.

[24] Harrolle MG, Floyd MF, Casper JM, Kelley KE, Bruton CM (2017). Physical activity constraints among Latinos. J Leis Res.

[25] Marcus BH, Dunsiger SI, Pekmezi DW, Larsen BA, Bock BC, Gans KM, Marquez B, Morrow KM, Tilkemeier P (2013). The Seamos Saludables study: a randomized controlled physical activity trial of Latinas. Am J Prev Med.

[26] Marcus BH, Hartman SJ, Larsen BA, Pekmezi D, Dunsiger SI, Linke S, Marquez B, Gans KM, Bock BC, Mendoza-Vasconez AS, Noble ML, Rojas C (2016). Pasos Hacia La Salud: a randomized controlled trial of an internet-delivered physical activity intervention for Latinas. Int J Behav Nutr Phys Act.

[27] Loya JC (2018). Systematic review of physical activity interventions in hispanic adults. Hisp Health Care Int.

[28] Larsen BA, Dunsiger S, Hartman S, Nodora J, Pekmezi DW, Marquez B, Noble M, Rojas C, Marcus BH (2014). Activo: assessing the feasibility of designing and implementing a physical activity intervention for Latino men. Int J Men's Health.

[29] Hartman SJ, Dunsiger SI, Bock BC, Larsen BA, Linke S, Pekmezi D, Marquez B, Gans KM, Mendoza-Vasconez AS, Marcus BH (2017). Physical activity maintenance among Spanish-speaking Latinas in a randomized controlled trial of an Internet-based intervention. J Behav Med.

[30] Gans KM, Dulin A, Palomo V, Benitez T, Dunsiger S, Dionne L, Champion G, Edgar R, Marcus B (2021). A tailored web- and text-based intervention to increase physical activity for Latino men: protocol for a randomized controlled feasibility trial. JMIR Res Protoc.

[31] Prochaska JO, Redding CA, Evers KE, Glanz K, Rimer BK, Viswanath K (2015). The transtheoretical model and stages of change. Health Behavior: Theory, Research, and Practice.

[32] Hayden-Wade HA, Coleman KJ, Sallis JF, Armstrong C (2003). Validation of the telephone and in-person interview versions of the 7-day PAR. Med Sci Sports Exerc.

[33] Lee S-Y, Stucky BD, Lee JY, Rozier RG, Bender DE (2010). Short Assessment of Health Literacy-Spanish and English: a comparable test of health literacy for Spanish and English speakers. Health Serv Res.

[34] Bandura A (1986). Social Foundations of Thought and Action: A Social Cognitive Theory.

[35] Freedson PS, Melanson E, Sirard J (1998). Calibration of the computer science and applications, inc. accelerometer. Med Sci Sports Exerc.

[36] White DK, Gabriel KP, Kim Y, Lewis CE, Sternfeld B (2015). Do short spurts of physical activity benefit cardiovascular health? The CARDIA study. Med Sci Sports Exerc.

[37] Piercy KL, Troiano RP, Ballard RM, Carlson SA, Fulton JE, Galuska DA, George SM, Olson RD (2018). The physical activity guidelines for Americans. JAMA.

[38] Sallis JF, Haskell WL, Wood PD, Fortmann SP, Rogers T, Blair SN, Paffenbarger RS Jr (1985). Physical activity assessment methodology in the five-city project. Am J Epidemiol.

[39] Blair SN, Haskell WL, Ho P, Paffenbarger RS Jr, Vranizan KM, Farquhar JW, Wood PD (1985). Assessment of habitual physical activity by a seven-day recall in a community survey and controlled experiments. Am J Epidemiol.

[40] Prince SA, Adamo KB, Hamel ME, Hardt J, Connor Gorber S, Tremblay M (2008). A comparison of direct versus self-report measures for assessing physical activity in adults: a systematic review. Int J Behav Nutr Phys Act.

[41] Leenders NY, Sherman WM, Nagaraja HN, Kien CL (2001). Evaluation of methods to assess physical activity in free-living conditions. Med Sci Sports Exerc.

[42] Dunn AL, Garcia ME, Marcus BH, Kampert JB, Kohl HW, Blair SN (1998). Six-month physical activity and fitness changes in Project Active, a randomized trial. Med Sci Sports Exerc.

[43] Mujahid MS, Diez Roux AV, Morenoff JD, Raghunathan T (2007). Assessing the measurement properties of neighborhood scales: from psychometrics to ecometrics. Am J Epidemiol.

[44] California Health Interview Survey 2013-2014 questionnaire topics. UCLA Center for Health Policy Research.

[45] Schuck AM, Rosenbaum DP, Hawkins DF (2008). The influence of race/ethnicity, social class, and neighborhood context on residents' attitudes toward the police. Police Q.

[46] Schuck AM, Rosenbaum DP (2005). Global and neighborhood attitudes toward the police: differentiation by race, ethnicity and type of contact. J Quant Criminol.

[47] Dulin-Keita A, Hannon L, Buys D, Casazza K, Clay O (2016). Surrounding community residents' expectations of HOPE VI for their community, health and physical activity. J Community Pract.

[48] Odgers CL, Caspi A, Bates CJ, Sampson RJ, Moffitt TE (2012). Systematic social observation of children's neighborhoods using Google Street View: a reliable and cost-effective method. J Child Psychol Psychiatry.

[49] Kelly CM, Wilson JS, Baker EA, Miller DK, Schootman M (2013). Using Google Street View to audit the built environment: inter-rater reliability results. Ann Behav Med.

[50] Hoehner CM, Ivy A, Ramirez LK, Handy S, Brownson RC (2007). Active neighborhood checklist: a user-friendly and reliable tool for assessing activity friendliness. Am J Health Promot.

[51] (2011). Navigating GOOGLE street view: a guide to conducting audits of the built environment using GOOGLE street view. The Robert Wood Johnson Foundation.

[52] Wilson JS, Kelly CM, Schootman M, Baker EA, Banerjee A, Clennin M, Miller DK (2012). Assessing the built environment using omnidirectional imagery. Am J Prev Med.

[53] Marcus BH, Selby VC, Niaura RS, Rossi JS (1992). Self-efficacy and the stages of exercise behavior change. Res Q Exerc Sport.

[54] Bock BC, Marcus BH, Pinto BM, Forsyth LH (2001). Maintenance of physical activity following an individualized motivationally tailored intervention. Ann Behav Med.

[55] Marcus BH, Rossi JS, Selby VC, Niaura RS, Abrams DB (1992). The stages and processes of exercise adoption and maintenance in a worksite sample. Health Psychol.

[56] Sallis JF, Grossman RM, Pinski RB, Patterson TL, Nader PR (1987). The development of scales to measure social support for diet and exercise behaviors. Prev Med.

[57] Cohen S, Kamarck T, Mermelstein R (1983). A global measure of perceived stress. J Health Soc Behav.

[58] Norris AE, Ford K, Bova CA (2016). Psychometrics of a brief acculturation scale for Hispanics in a probability sample of urban Hispanic adolescents and young adults. Hispanic J Behav Sci.

[59] Garcia DO, Valdez LA, Aceves B, Bell ML, Humphrey K, Hingle M, McEwen M, Hooker SP (2019). A gender- and culturally sensitive weight loss intervention for Hispanic men: results from the Animo pilot randomized controlled trial. Health Educ Behav.

[60] Larsen BA, Benitez TJ, Mendoza-Vasconez AS, Hartman SJ, Linke SE, Pekmezi DJ, Dunsiger SI, Nodora JN, Gans KM, Marcus BH (2020). Randomized trial of a physical activity intervention for Latino men: Activo. Am J Prev Med.

[61] Frediani JK, Bienvenida AF, Li J, Higgins MK, Lobelo F (2020). Physical fitness and activity changes after a 24-week soccer-based adaptation of the U.S diabetes prevention program intervention in Hispanic men. Prog Cardiovasc Dis.

[62] Mclaughlin M, Delaney T, Hall A, Byaruhanga J, Mackie P, Grady A, Reilly K, Campbell E, Sutherland R, Wiggers J, Wolfenden L (2021). Associations between digital health intervention engagement, physical activity, and sedentary behavior: systematic review and meta-analysis. J Med Internet Res.

[63] Linke SE, Dunsiger SI, Gans KM, Hartman SJ, Pekmezi D, Larsen BA, Mendoza-Vasconez AS, Marcus BH (2019). Association between physical activity intervention website use and physical activity levels among Spanish-speaking Latinas: randomized controlled trial. J Med Internet Res.

[64] Marquez DX, McAuley E (2006). Social cognitive correlates of leisure time physical activity among Latinos. J Behav Med.

[65] Laffrey SC (2000). Physical activity among older Mexican American women. Res Nurs Health.

[66] Laffrey SC, Asawachaisuwikrom W (2001). Development of an exercise self-efficacy questionnaire for older Mexican American women. J Nurs Meas.

[67] Freedland KE (2020). Pilot trials in health-related behavioral intervention research: problems, solutions, and recommendations. Health Psychol.

